# Uptake, positivity, and equity of online postal self-sampling for chlamydia testing in England: a retrospective cohort study

**DOI:** 10.1016/j.lanepe.2025.101412

**Published:** 2025-08-15

**Authors:** Alison Howarth, Ana Harb, Hamish Mohammed, Fiona Burns, Claudia Estcourt, Sonja C.M. Bloch, Andrew Copas, Jonathan O'Sullivan, Tamilore Sonubi, Oliver Stirrup, Anna Tostevin, John Saunders, Jo Gibbs

**Affiliations:** a UCL Institute for Global Health, Mortimer Market Centre, off Capper Street, London, WC1E 6JB, UK; b Blood Safety, Hepatitis, STIs and HIV Division, UK Health Security Agency, 61 Colindale Avenue, London, NW9 5EQ, UK; c School of Health and Life Sciences, Glasgow Caledonian University, Cowcaddens, Glasgow, G4 0BA, UK; d Islington Council, 222 Upper Street, London, N1 1XR, UK

**Keywords:** Chlamydia, STIs, Online STI testing, STI service provision, Digital health, Public health

## Abstract

**Background:**

Chlamydia is the most commonly reported sexually transmitted infection (STI) in Europe and untreated chlamydia is associated with poor health outcomes. Online postal self-sampling enables people to test for STIs including chlamydia without having to visit a health-care provider, but the extent to which the addition of this mode of testing in England has impacted access to testing in different populations is unclear. In England, there is national-level surveillance data enabling identification of the factors associated with use of online postal self-sampling (OPSS) for chlamydia testing. The aim of this analysis was to determine the change in chlamydia testing, chlamydia positivity, and test location as a result of the introduction of OPSS, and to determine socio-demographic factors associated with uptake of OPSS services compared to testing in-person.

**Methods:**

We conducted a retrospective cohort study analysing data on all publicly funded chlamydia tests between 01/01/2015 and 31/12/2022 in England using two pseudonymised national surveillance systems (GUMCAD STI Surveillance System and CTAD Chlamydia Surveillance System) for STIs. We conducted a descriptive analysis of 25,171,919 chlamydia tests to establish the uptake and positivity of chlamydia tests by testing mode and gender over time. We used bivariate and multivariable logistic regression to examine associations of uptake of testing and positivity of tests with sociodemographic characteristics and testing by OPSS or a different mode.

**Findings:**

The overall number of chlamydia tests/quarter (OPSS and in-person) gradually increased over time until 2019 (884,843 tests/quarter in quarter 1) and then declined in early 2020 (376,118 in quarter 2) and had not returned to 2019 levels by the end of 2022 (715,166 in quarter 4). During this time, the proportion of OPSS testing completed through OPSS increased from 2.6% (88,144/3,433,987) in 2015 to 38.4% (1,168,828/2,972,226) in 2022. Women were less likely than men to use OPSS compared to all available in-person testing (aOR = 0.75, 95% CI 0.75–0.75)). Those aged 20–24 were more likely to use OPSS than 15–19-year-olds (aOR = 1.55, 95% CI 1.55–1.56) and use of OPSS then decreased with increasing age. People in the most deprived areas were the least likely to use OPSS (aORs 1.18–1.28 for index of multiple deprivation quintile groups 2–5 vs 1). People were less likely to test positive using OPSS compared to in-person testing (aOR = 0.87, 95% CI 0.87–0.88). Between 2015 and 2022 OPSS chlamydia test-positivity decreased from 9.3% (2551/27,557) to 7.5% (34,050/454,596) in men and 7.4% (4458/60,367) to 6.1% (43,088/702,867) in women. During the same period, chlamydia test-positivity in sexual health services increased from 8.2% (57,139/692,873) to 10.6% (43,061/406,161) in men and 6.4% (51,080/797,143) to 7.9% (33,292/420,760) in women.

**Interpretation:**

We have found changes in access to care, with a shift towards testing via OPSS, and variations in who tests where and differences in positivity by testing mode and gender. Further research is needed to ensure available testing pathways meet the needs of all populations.

**Funding:**

10.13039/501100000272National Institute for Health and Care Research.

**Research in context**

**Evidence before this study**

We searched for studies on the impact of online postal self-sampling on access to care and clinical and public health outcomes. Our scoping review of the literature covered the evidence up to July 2021, and we searched PubMed and Web of Science without language restrictions from July 2021 to February 2025. Search terms were “STI” OR “Sexually Transmitted Disease” AND “home-based” OR “self-sampling” OR “online” or “digital” and searched within these articles for those referencing in-person services. The evidence indicates that, since the expansion of OPSS in 2015, evaluations have been based on data from single sites, self-sampling providers, or a limited time period. To our knowledge, this is the first study to analyse data on all publicly-funded chlamydia testing in England since the expansion of OPSS in 2015 to identify any inequities resulting from the reduction in access to in-person testing and uptake of OPSS over the course of its roll out.

**Added value of this study**

We conducted an analysis of the national evidence in England, including data from 25 million chlamydia tests over a period of eight years. The number and proportion of OPSS tests continued to increase over the eight-year study period while OPSS test positivity tended to decrease. Contrary to previous studies, which have focused on comparing OPSS with sexual health services only, we found that women were proportionately less likely than men to use OPSS compared to all other in-person testing. The findings from our study were consistent with previous single site studies showing that the youngest age groups and people living in more deprived areas are less likely to access OPSS.

**Implications of all the available evidence**

We found differences in chlamydia positivity by testing mode, and variation in use of OPSS by gender, age group and area level deprivation. Our findings demonstrate that different modes of testing are needed to optimise access across all population groups. It is important to ensure that reducing in-person testing does not increase unmet need, although the data suggest that appropriate channelling to OPSS is occurring for those at less risk. Further research is needed to explore the cost-effectiveness of OPSS and its contribution to equitable sexual healthcare.

## Introduction

Chlamydia is the most commonly reported bacterial sexually transmitted infection (STI) in Europe, with a 13% increase in incidence between 2014 and 2022.^1^ In England, numbers of positive diagnoses rose from 160,279 in 2021 to 199,233 in 2022, an increase of 24%,^2^ and remained at this level in 2023.^3^ Undiagnosed and untreated chlamydia is associated with poor health outcomes, particularly among women, in whom it may lead to complications including pelvic inflammatory disease, tubal factor infertility and ectopic pregnancy.^4^^,^^5^ Although early diagnosis and treatment can prevent development of these complications,^6^^,^^7^ most chlamydia infections are asymptomatic and may therefore go undetected without screening. To address this, the National Chlamydia Screening Programme (NCSP) was fully implemented in England in 2008.^8^ Originally targeted at all genders aged under 25, since 2021 the NCSP has focused on reducing the reproductive morbidity associated with untreated chlamydia and now only recommends opportunistic screening outside sexual health services to people assigned female at birth (including cisgender women, trans men and non-binary people).^8^ The British Association for Sexual Health and HIV (BASHH) recommends asymptomatic STI testing at the start of a relationship, annually and after partner change for those who are sexually active, and on a 3 monthly basis for those who are at higher risk of STIs (e.g., pre-exposure prophylaxis for HIV users and those with multiple or anonymous partners).^9^

Over the past 15 years, access to home-based screening for chlamydia has become increasingly widespread in Europe.^10^^,^^11^ In England, people can access free in-person chlamydia testing at sexual health services (SHSs), primary care (general practice and pharmacies) and a range of other in-person services. Online postal self-sampling (OPSS) seeks to address the growing demand for STI testing through provision of free STI self-sampling kits which people order online for home delivery or collection from a SHS or pharmacy. People complete an online consultation to determine eligibility for remote testing and which test kit they require. If eligible, a kit is posted to their home, or they collect it from a SHS or pharmacy. They return their samples by post to a laboratory for processing and receive their results via SMS or access them online. If people report symptoms during the online consultation, they may not be offered a self-sampling kit—this differs between regions, is dependent on type of symptom in some areas, and has changed over time. STIs tested for, and types of specimens collected, in OPSS services also varies between regions and has changed over time but mainly aligns with national testing guidance.^9^

In England, the past decade has seen an active drive to channel asymptomatic people from testing in SHSs towards OPSS in some areas.^12^ OPSS services expanded from 2015 onwards, and at the same time the national chlamydia surveillance system (CTAD) began capturing online testing. Analysis of national surveillance data for chlamydia testing among young women aged 15–24 years shows a yearly increase in use of OPSS (2018–2022) despite a drop in overall testing at the start of the COVID-19 pandemic in 2020.^2^ Our final year (2022) marks the beginning of the post-pandemic period although service delivery was further disrupted by the Mpox outbreak, with the first case being identified in May 2022.^13^

An early comparison (2006–2010) of chlamydia test positivity among young people found OPSS positivity (7.6%) to be higher than test positivity in general practice (5.6%), and comparable to testing in community sexual and reproductive health services (8.2%).^14^ Previous studies, however, have found people testing in SHSs were more likely to test positive for chlamydia or gonorrhoea than those who tested using OPSS in London (14.4% vs 4.4%)^15^ and in Birmingham (10% vs 8%).^16^

Although OPSS may increase total testing activity and free up in-person SHSs for more complex cases,^17^ it is paramount that equitable access to sexual healthcare is maintained for all. Initial evidence from observational data from single services suggests OPSS is accessed by a higher proportion of women, those living in less deprived areas and people of white ethnicity.^18^^,^^19^ Return rates for STI kits vary^15^^,^^20^, ^21^, ^22^ with non-return more likely among heterosexual men, those who are symptomatic, and those in more deprived areas.^17^

The aim of this analysis was to understand the factors associated with use of different chlamydia testing options, since this is required to ensure equitable access to sexual healthcare for all. We examined use of OPSS and in-person services for all chlamydia testing conducted in England over an eight-year period (2015–2022) and compared chlamydia test positivity for different testing settings over time.

## Methods

This analysis was undertaken to address primary objectives of both ASSIST (www.assist-study.org), a mixed-methods realist evaluation of online postal self-sampling,^23^ and SEQUENCE digital, a programme of research to optimise, trial and evaluate an eSexual Health Clinic (www.sequencedigital.org.uk).

### Study design and data sources

We conducted a retrospective cohort study of chlamydia testing activity among people in England between 1st January 2015 to 31 December 2022, analysing data from the national GUMCAD STI Surveillance System^24^ and the CTAD Chlamydia Surveillance System.^25^ In combination, these pseudonymised datasets provide the best possible coverage of different services providing chlamydia testing in England. The datasets are collected by the UK Health Security Agency (UKHSA) and over 99% of reporters to both datasets submitted data for all years in the analysis, with the exception of 2020.^26^ GUMCAD is a patient-level dataset which includes depersonalised information on all attendances at SHSs in England. It is a comprehensive source of data on people accessing those services but is limited in its degree of representativeness to the general population. CTAD contains depersonalised information from primary diagnostic laboratories on all publicly funded chlamydia tests and diagnoses, including data from non-specialist STI-related care such as general practice and pharmacies, so is a reliable source of data for people being tested for chlamydia. Neither dataset has data on people accessing STI testing from private health providers, but this is a very small market in England. Both GUMCAD and CTAD collect data from specialist SHSs. However, the data from specialist SHSs collected by laboratories for CTAD does not have information on area of residence of the patient and therefore these data are dropped and replaced with the data from specialist SHSs collected in GUMCAD. Only one test or diagnosis for each unique person identifier is counted within a 6-week episode to avoid double counting.^27^ There is a high level of completion of most demographic variables in both datasets, with the exception of ethnicity in CTAD.^26^

Key events that impacted delivery of and access to chlamydia testing in England between 2015 and 2022 are summarised in Panel 1.

Panel 1 **Key events impacting chlamydia testing provision in England from 2015 to 2022**

**2015**

Beginning of expansion of Online Postal Self-Sampling (OPSS) services, both in terms of size of provision in existing services and number of services providing OPSS, across England following the Health and Social Care Act 2012.

**2018**

OPSS to be incorporated into service specifications according to English national guidance

Roll out of London's OPSS service Sexual Health London (SHL) begins in January expanding to cover all London boroughs except Croydon, Greenwich and Hillingdon.

**2020**

First national lockdown (late March 2020–June 2020).

- introduction of policy to test only symptomatic cases in sexual health services with significant restriction in access to face-to-face appointments and in person testing, and a shift to remote service delivery
- introduction of testing for those reporting non-urgent symptoms via some OPSS services

Local lockdowns (September 2020–November 2020).

**2021**

Second national lockdown (January 2021–July 2021).

- return to significant restriction on face-to-face appointments and in person testing, and a shift back to more activity occurring through remote service delivery

Change in the National Chlamydia Screening Programme from offering opportunistic screening to all sexually active young people aged 15–24 years to focus on those assigned female at birth.

**2022**

Mpox outbreak impacts sexual health services in London for several months

### Statistical analysis

The analysis included England residents aged 15 years and older at the time of chlamydia testing or diagnosis. England is divided into small geographical areas known as lower super output areas (LSOAs), a standard statistical geography used by the Office for National Statistics.^28^ These areas were matched to quintiles of the 2015 and 2019 Indices of Multiple Deprivation (IMD) dataset^29^ to provide a measure of area-level socioeconomic deprivation. LSOAs were also matched to the 2011 census area classification^28^ to categorise living in an urban or rural setting or living in London. We created a separate category for London, rather than including it within the urban category as, due to the high volume of testing activity in London, any associations found are likely representative of London but could miss nuances in other urban areas.

We excluded tests where the age group was unknown as we do not think age group is missing at random and is more likely due to systematic errors in data collection and reporting. We included the following demographic characteristics: age group; gender; ethnic group (self-reported and based on how a person identifies as categorised using the national Census classification); residential area-level deprivation (defined by IMD quintile, where 1 is the most deprived and 5 is the least deprived); UKHSA public health region of residency; and area of residency (rural, London, other urban). In GUMCAD, gender is primarily self-reported by service users in clinical services. CTAD is reported by laboratories from various clinical and non-clinical venues, so the reporting practice in those settings might be different. Gender has therefore not been disaggregated beyond men including trans men and women including trans women. Sexual orientation was not considered in the analysis as these are not captured in the CTAD dataset. We included the following clinical characteristics: year of testing; testing mode and diagnosis. In-person testing modes, which are all testing modes listed in Table 1 except OPSS, were combined to compare in-person testing with OPSS.

All analyses were carried out using Stata 17.0 (StataCorp LP, College Station, TX, USA). The Pearson's chi-square test was used to test whether characteristics (demographic and clinical) differ between men and women testing for chlamydia, and between those testing by OPSS or another mode. We used logistic regression to determine the crude associations between characteristics and testing via OPSS vs in-person, and associations adjusted for all other characteristics. The full area of residency variable was used in unadjusted analysis to highlight any differences between London and other urban areas, and rural vs urban was used in multivariable analysis due to collinearity with region of residency. We conducted sensitivity analyses including ethnicity, and the bias due to the high degree of item non-response for this variable become increasingly apparent in the measures of association for several key covariates. We therefore elected to do the regression analysis without ethnicity and have excluded those results from this paper. However, there is evidence of a very strong correlation between ethnicity and socioeconomic deprivation (especially for STI diagnoses) and having controlled for deprivation in our regression analysis, we can therefore make inferences about the equity of OPSS by ethnic group.

Our main analyses use data from 2015 to 2022 but in secondary analysis we also explore the association between characteristics and OPSS compared to in-person testing in 2022 alone, and this 2022 analysis is repeated for OPSS compared to SHS testing. We also use logistic regression to determine how characteristics are associated with test positivity (i.e. diagnosis of chlamydia) between 2015 and 2022 unadjusted and adjusted for all other characteristics.

We focused the main analysis on OPSS vs in person due to the ASSIST and SEQUENCE Digital research objectives, and the ethical approval in place. We conducted the analysis OPSS vs SHS testing for the same reason and because of the large proportion of testing that has traditionally occurred in SHS in England, with SHS and OPSS comprising ∼65% of all chlamydia tests in the NCSP target group.^2^

As neither CTAD nor GUMCAD capture personal identifiers, it is not possible to link individuals between testing episodes at different services (such as between in-person and OPSS services). We therefore did not account for correlation between testing episodes within any given individual within our main analysis. However, as a sensitivity analysis, we also fitted our logistic regression models of OPSS vs. in person testing using generalized estimating equations (GEE) with exchangeable working correlation to acknowledge repeat in-person tests within individuals at the same service over time.

### Ethics statement

Ethical approval was granted by the NHS South Central–Berkshire B Research Ethics Committee (21/SC/0223). This analysis was undertaken for health protection purposes under the permissions granted to the UK Health Security Agency (UKHSA) to collect and process pseudonymised CTAD and GUMCAD surveillance data under Regulation 3 of The Health Service (Control of Patient Information) Regulations 2020 and under Section 251 of the National Health Service (NHS) Act 2006. Informed consent was therefore not required.

### Role of the funding source

The funder of the study had no role in study design, data collection, data analysis, data interpretation, or writing of the report.

## Results

A total of 25,171,919 chlamydia tests were included in this analysis. Two thirds of tests were undertaken by women (66.9% (16,839,034/25,171,919)) and one third by men (31.7% (7,974,151/25,171,919) (Table 1). The largest proportion of chlamydia testing was undertaken in SHSs (40.2% (10,108,131/25,171,919)), whilst 23.5% (5,908,492/25,171,919) of tests were accessed in general practice and 17.5% (4,408,869/25,171,919) via OPSS.

**Table 1:** Characteristics of those testing for chlamydia, by gender (2015–22).

	All%	Gender^b^	
		Men %^a^, ^c^	Women %^a^, ^c^
	25,171,919	7,974,151	16,839,034
**Background information**			
**Gender [*N*]**			
Men^c^	7,974,151 (31.7)	–	–
Women^c^	16,839,034 (66.9)	–	–
Other/Unknown	358,734 (1.4)	–	–
**Age group**			
15–19 years	3,138,192 (12.5)	762,826 (9.6)	2,316,777 (13.8)
20–24 years	6,730,227 (26.7)	2,006,960 (25.2)	4,622,220 (27.4)
25–34 years	8,846,591 (35.1)	2,913,937 (36.5)	5,815,794 (34.5)
35–44 years	4,022,392 (16.0)	1,299,430 (16.3)	2,673,793 (15.9)
45–64 years	2,274,291 (9.0)	901,667 (11.3)	1,342,735 (8.0)
65 years & over	160,226 (0.6)	89,331 (1.1)	67,715 (0.4)
**Ethnic group**^a^			
Asian–Bangladeshi	74,010 (0.3; 0.5)	29,835 (0.4; 0.5)	42,986 (0.3; 0.4)
Asian–Indian	301,988 (1.2; 1.8)	127,518 (1.6; 2.1)	170,780 (1.0; 1.7)
Asian–Pakistani	208,635 (0.8; 1.3)	88,599 (1.1; 1.4)	116,993 (0.7; 1.1)
Asian–Chinese	114,560 (0.5; 0.7)	49,199 (0.6; 0.8)	64,060 (0.4; 0.6)
Asian–other	231,198 (0.9; 1.4)	100,435 (1.3; 1.6)	127,443 (0.8; 1.2)
Black–African	771,144 (3.1; 4.7)	330,443 (4.1; 5.4)	429,005 (2.5; 4.2)
Black–Caribbean	640,296 (2.5; 3.9)	258,582 (3.2; 4.2)	375,241 (2.2; 3.7)
Black–other	201,834 (0.8; 1.2)	82,598 (1.0; 1.3)	116,295 (0.7; 1.1)
Mixed	839,162 (3.3; 5.1)	314,266 (3.9; 5.1)	512,325 (3.0; 5.0)
White–British	10,894,612 (43.3; 65.7)	3,822,941 (47.9; 61.9)	6,898,959 (41.0; 67.9)
White–Irish	169,559 (0.7; 1.0)	84,015 (1.1; 1.4)	83,685 (0.5; 0.8)
White–Other	1,823,188 (7.2; 11.0)	760,949 (9.5; 12.3)	1,041,700 (6.2; 10.2)
Other ethnic groups	312,843 (1.2; 1.9)	131,309 (1.6; 2.1)	177,401 (1.1; 1.7)
Unknown	8,588,890 (34.1; -)	1,793,462 (22.5; -)	6,682,161 (39.7; -)
**IMD**^d^			
1—most deprived	5,470,769 (21.7)	1,643,413 (20.6)	3,740,741 (22.2)
2	6,032,494 (24.0)	2,008,110 (25.2)	3,945,736 (23.4)
3	5,067,826 (20.1)	1,628,371 (20.4)	3,369,288 (20.0)
4	4,106,988 (16.3)	1,287,562 (16.1)	2,764,694 (16.4)
5—least deprived	3,642,786 (14.5)	1,088,437 (13.6)	2,503,563 (14.9)
Unknown	851,056 (3.4)	318,258 (4.0)	515,012 (3.1)
**Region of residency**			
London	6,858,584 (27.2)	2,747,300 (34.5)	4,046,662 (24.0)
North East	1,028,762 (4.1)	278,864 (3.5)	734,995 (4.4)
North West	2,972,657 (11.8)	869,737 (10.9)	2,020,855 (12.0)
Yorkshire & Humber	2,368,262 (9.4)	600,249 (7.5)	1,756,321 (10.4)
East Midlands	1,916,118 (7.6)	493,747 (6.2)	1,397,094 (8.3)
East of England	2,339,158 (9.3)	684,743 (8.6)	1,612,362 (9.6)
West Midlands	2,001,552 (8.0)	577,579 (7.2)	1,394,478 (8.3)
South East	3,195,993 (12.7)	966,923 (12.1)	2,178,648 (12.9)
South West	2,162,879 (8.6)	608,941 (7.6)	1,527,062 (9.1)
Unknown	327,954 (1.3)	146,068 (1.8)	170,557 (1.0)
**London-rural-urban**			
Rural	2,529,140 (10.0)	707,152 (8.9)	1,787,300 (10.6)
London	6,853,972 (27.2)	2,745,810 (34.4)	4,043,574 (24.0)
Other urban	15,065,474 (59.9)	4,258,013 (53.4)	10,565,167 (62.7)
Unknown	723,333 (2.9)	263,176 (3.3)	442,993 (2.6)
**Testing information**			
**Year of testing**			
2015	3,460,881 (13.7)	1,058,097 (13.3)	2,375,890 (14.1)
2016	3,329,283 (13.2)	1,027,410 (12.9)	2,262,954 (13.4)
2017	3,182,064 (12.6)	992,138 (12.4)	2,159,760 (12.8)
2018	3,310,084 (13.1)	1,070,564 (13.4)	2,208,668 (13.1)
2019	3,463,456 (13.8)	1,131,780 (14.2)	2,292,567 (13.6)
2020	2,508,722 (10.0)	787,560 (9.9)	1,692,785 (10.1)
2021	2,843,026 (11.3)	886,984 (11.1)	1,893,802 (11.2)
2022	3,074,403 (12.2)	1,019,618 (12.8)	1,952,608 (11.6)
**Testing mode**			
Sexual health services	10,108,131 (40.2)	4,584,689 (57.5)	5,288,080 (31.4)
OPSS	4,408,869 (17.5)	1,631,806 (20.5)	2,749,444 (16.3)
Community	1,166,585 (4.6)	330,957 (4.2)	811,687 (4.8)
GP	5,908,492 (23.5)	641,926 (8.1)	5,246,861 (31.2)
Pharmacy	108,957 (0.4)	27,044 (0.3)	81,041 (0.5)
TOP services^e^	313,766 (1.2)	610 (0.0)	306,126 (1.8)
Other	2,809,749 (11.2)	709,183 (8.9)	2,065,003 (12.3)
Unknown	347,370 (1.4)	47,936 (0.6)	290,792 (1.7)
**Positive diagnosis**			
No	23,601,836 (93.8)	7,296,490 (91.5)	15,975,468 (94.9)
Yes	1,570,083 (6.2)	677,661 (8.5)	863,566 (5.1)

a Where missing data are >5%, proportion of tests is shown including *and* excluding unknown cases (including; excluding).

b All associations between gender and the demographics were calculated using chi-square (data not shown) and were significant at p < 0.0001.

c Men including trans men, women including trans women.

d IMD, Index of Multiple deprivation according to postcode of residence.

e TOP, Termination of Pregnancy.

The distribution of chlamydia testing for age group, IMD quintile and year of testing was broadly similar between genders. The proportion of missing data for ethnic group was higher for women (39.7% (6,682,161/16,839,034)) than men (22.5% (1,1793,462/7,974,151) (p < 0.0001). Among those tested, men were more likely to be London residents than women (34.5% (2,747,300/7,974,151) vs 24.0% (4,043,574/16,839,034)) (p < 0.0001). A higher proportion of men than women tested in SHSs (57.5% (4,584,689/7,974,151) vs 31.4% (5,288,080/16,839,034) (p < 0.0001) and women were more likely to test in general practice (31.2% (5,246,861/16,839,034 vs 8.1% (641,926/7,974,151) (p < 0.0001)). Men were more likely than women to test positive for chlamydia (8.5% (677,661/7,974,151) vs 5.1% (863,566/16,839,034) p < 0.001)).

**Fig. 1:**
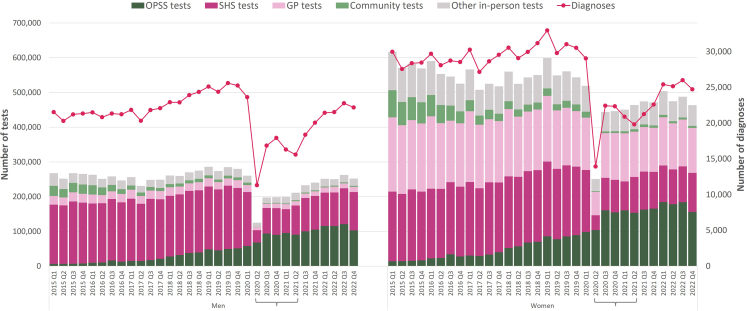
**Mode of chlamydia testing and diagnosis, by gender and year**. Brackets along the X axis indicate: The first national lockdown (late March 2020–June 2020) occurred in 2020 Q2; local lockdowns (September 2020–November 2020) occurred during 2020 Q3 to 2020 Q4; and the second national lockdown (January 2021–July 2021) occurred mainly in 2021 Q1 and 2021 Q2.

Fig. 1 describes the use of OPSS, SHS, general practice (GP), community and other in-person settings for chlamydia testing for each quarter from 2015 to 2022. There was a drop in testing across all in-person settings for men and women after the first UK lockdown (23 March 2020). In-person testing then increased from the third quarter of 2020 and remained stable, but had not recovered to pre-pandemic levels by the last quarter of 2022. There was a gradual overall increase in OPSS testing for men and women from 2015 to the first quarter of 2020. The first UK lockdown was followed by a step up in OPSS testing and subsequent gradual increase before dropping in the last quarter of 2022. In men and women, the proportion of OPSS testing increased from 2.6% (87,924/3,433,987) in 2015 to 38.9% (1,157,463/2,972,226) in 2022 while the proportion of SHS testing fell from 43.4% (1,490,016/3,433,987) to 27.8% (826,921/2972,226) (see Fig. 1 and Appendix 1).

The pre-pandemic trends for overall diagnoses were different for men and women (Fig. 1). Positive diagnoses among men increased from 21,537 in the first quarter of 2015 to 25,253 in the last quarter of 2019 whereas diagnoses among women remained comparatively stable during this period (from 29,983 to 30,528). Diagnoses dropped in the second quarter of 2020 (corresponding to the first UK lockdown) to a low of 11,308 for men and 13,919 for women. The subsequent trend is similar for men and women with the second lowest number of diagnoses in the second quarter of 2021 for men (15,599) and women (19,819).

**Fig. 2:**
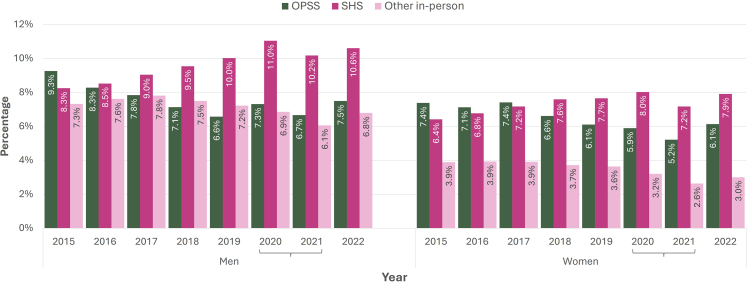
**Chlamydia test positivity, by mode^§^, gender and year**. ^§^ Other in-person testing services include community sexual and reproductive health services, general practice, pharmacy, termination of pregnancy and other free in-person testing services. Brackets along the X axis indicate years disrupted by COVID-19.

The trends in test positivity across different settings were similar for men and women (Fig. 2). The proportion of OPSS tests with a positive result declined from 9.3% (2551/27,557) in 2015 to 7.5% (34,050/454,596) in 2022 among men and from 7.4% (4458/60,367) to 6.1% (43,088/702,867) among women, while the proportion of SHS tests with a positive result increased from 8.3% (57,139/692,873) to 10.6% (43,061/406,161) among men and 6.4% (51,080/797,143) to 7.9% (33,292/420,760) among women. For both men and women, the highest SHS test positivity was in 2020 (11.0% 37,534/341,215) and 8.0% (32,632/407,903), respectively)—which is when Fig. 1 shows an overall decrease in diagnoses and in SHS testing (see also Appendix 1).

### OPSS vs all in-person chlamydia testing

Comparing use of OPSS and all in-person chlamydia testing services, multivariable logistic regression indicated women were less likely than men to use OPSS (adjusted odds ratio (aOR) 0.75 [confidence interval: 0.75–0.75]) (Table 2). Compared to the youngest age group (15–19 years), 20–24-year-olds were more likely to use OPSS (aOR 1.55 [1.55–1.56]) whereas older age groups were increasingly less likely to use OPSS. People living in the most deprived areas were the least likely to use OPSS, whereas those living in London were more likely to use OPSS than all other areas. In unadjusted analysis, use of OPSS was far greater in London compared to rural areas (OR 1.74 [1.73–1.75]), but there was little difference between rural areas and urban areas outside London (OR 0.98 [0.98–0.99]). Chlamydia tests were increasingly likely to have been accessed via OPSS over time with an aOR of 27.05 [26.86–27.25] in 2022 compared to 2015. The GEE sensitivity analysis of mode of testing by sociodemographic factors and testing behaviour (2015–2022) did not show any substantial differences in results, and the findings of this analysis can be found in Appendix 2.

**Table 2:** Mode of testing (OPSS vs in-person) by sociodemographic factors and testing behaviour (2015–22).

	N of tests	In-person %	OPSS %^d^	OR^d^	[CI]	aOR^d^	[CI]
**Gender**^a^							
Men	7,926,215	6,294,409 (79.4)	1,631,806 (20.6)	Ref		Ref	
Women	16,548,242	13,798,798 (83.4)	2,749,444 (16.6)	0.77	[0.77–0.77]	0.75	[0.75–0.75]
Other/Unknown	350,092	322,473 (92.1)	27,619 (7.9)	–	–	–	–
**Age group**							
15–19 years	3,090,817	2,630,050 (85.1)	460,767 (14.9)	Ref		Ref	
20–24 years	6,655,847	5,038,471 (75.7)	1,617,376 (24.3)	1.83	[1.82–1.84]	1.55	[1.55–1.56]
25–34 years	8,727,830	7,101,988 (81.4)	1,625,842 (18.6)	1.31	[1.30–1.31]	0.82	[0.82–0.83]
35–44 years	3,954,544	3,465,305 (87.6)	489,239 (12.4)	0.81	[0.80–0.81]	0.46	[0.46–0.46]
45–64 years	2,238,058	2,032,608 (90.8)	205,450 (9.2)	0.58	[0.57–0.58]	0.33	[0.32–0.33]
65 years & over	157,453	147,258 (93.5)	10,195 (6.5)	0.40	[0.39–0.40]	0.20	[0.20–0.21]
**IMD**^b^							
1—most deprived	5,382,701	4,577,238 (85.0)	805,463 (15.0)	Ref		Ref	
2	5,948,222	4,811,457 (80.9)	1,136,765 (19.1)	1.34	[1.34–1.35]	1.18	[1.17–1.18]
3	5,010,645	4,068,197 (81.2)	942,448 (18.8)	1.32	[1.31–1.32]	1.20	[1.19–1.20]
4	4,052,141	3,309,907 (81.7)	742,234 (18.3)	1.27	[1.27–1.28]	1.28	[1.27–1.28]
5—least deprived	3,602,215	2,991,445 (83)	610,770 (17)	1.16	[1.16–1.16]	1.20	[1.19–1.20]
Unknown	828,625	657,436 (79.3)	171,189 (20.7)	–	–	–	–
**Region of residency**							
London	6,820,758	5,178,384 (75.9)	1,642,374 (24.1)	Ref		Ref	
North East	988,416	886,441 (89.7)	101,975 (10.3)	0.36	[0.36–0.37]	0.34	[0.33–0.34]
North West	2,865,396	2,582,481 (90.1)	282,915 (9.9)	0.35	[0.34–0.35]	0.34	[0.34–0.34]
Yorkshire & Humber	2,361,443	2,002,946 (84.8)	358,497 (15.2)	0.56	[0.56–0.57]	0.54	[0.54–0.55]
East Midlands	1,888,801	1,551,567 (82.1)	337,234 (17.9)	0.69	[0.68–0.69]	0.68	[0.67–0.68]
East of England	2,333,138	1,884,355 (80.8)	448,783 (19.2)	0.75	[0.75–0.75]	0.74	[0.74–0.75]
West Midlands	1,985,554	1,682,149 (84.7)	303,405 (15.3)	0.57	[0.57–0.57]	0.57	[0.57–0.57]
South East	3,100,684	2,631,448 (84.9)	469,236 (15.1)	0.56	[0.56–0.56]	0.54	[0.54–0.55]
South West	2,157,507	1,774,966 (82.3)	382,541 (17.7)	0.68	[0.68–0.68]	0.70	[0.69–0.70]
Unknown	322,852	240,943 (74.6)	81,909 (25.4)	–	–	–	–
**London-rural-urban**^c^							
Rural	2,487,943	2,104,217 (84.6)	383,726 (15.4)	Ref		Ref	
London	6,816,179	5,175,123 (75.9)	1,641,056 (24.1)	1.74	[1.73–1.75]	1.13	[1.12–1.13]
Other urban	14,818,621	12,563,027 (84.8)	2,255,594 (15.2)	0.98	[0.98–0.99]		
Unknown	701,806	573,313 (81.7)	128,493 (18.3)	–	–	–	–
**Year of testing**							
2015	3,416,842	3,328,698 (97.4)	88,144 (2.6)	Ref		Ref	
2016	3,268,821	3,110,384 (95.2)	158,437 (4.8)	1.92	[1.91–1.94]	1.92	[1.91–1.94]
2017	3,128,445	2,925,538 (93.5)	202,907 (6.5)	2.62	[2.60–2.64]	2.63	[2.61–2.66]
2018	3,273,977	2,887,124 (88.2)	386,853 (11.8)	5.06	[5.02–5.10]	5.12	[5.08–5.16]
2019	3,414,926	2,879,451 (84.3)	535,475 (15.7)	7.02	[6.97–7.07]	7.12	[7.07–7.17]
2020	2,469,708	1,639,427 (66.4)	830,281 (33.6)	19.13	[18.99–19.26]	20.26	[20.11–20.41]
2021	2,804,913	1,766,969 (63)	1,037,944 (37)	22.18	[22.03–22.34]	24.75	[24.57–24.93]
2022	3,046,917	1,878,089 (61.6)	1,168,828 (38.4)	23.50	[23.34–23.67]	27.05	[26.86–27.25]
**Positive diagnosis**							
No	23,267,663	19,143,739 (82.3)	4,123,924 (17.7)	Ref		Ref	
Yes	1,556,886	1,271,941 (81.7)	284,945 (18.3)	1.04	[1.04–1.04]	0.87	[0.87–0.88]

OR, odds ratio, CI, confidence intervals, aOR, adjusted odds ratio.

a Men including trans men, women including trans women. Tests with unknown testing service were excluded.

b IMD, Index of Multiple deprivation according to postcode of residence.

c The full area of residency variable was used in unadjusted analysis to highlight any differences between London and other urban areas, and rural vs urban (London and Other urban combined) was used in multivariable analysis due to collinearity with region of residency.

d All associations are significant at p < 0.0001.

By 2022, the proportion of OPSS had increased from 2.6% (88,144/3,416,842) in 2015 to 38.4% (1,168,828/3,046,917) of all chlamydia testing. However, the associations between sociodemographic characteristics and testing behaviour with use of OPSS remained largely the same (Appendix 3), with the same trends for gender, age group, IMD, London-urban-rural and test positivity. While London had the highest proportion of OPSS testing across the eight-year study period, in 2022 the highest proportion of OPSS use was in the East of England (50.9%) with London the second highest (49.4%).

In unadjusted analysis, OPSS was more likely to yield positive results but in adjusted analysis chlamydia testing using OPSS was associated with a lower test positivity, after adjusting for gender, age group, IMD, region of residency, rural-urban and year of testing (aOR 0.88 [0.87–0.88]). The findings of the analysis of chlamydia diagnosis by sociodemographic factors and testing behaviour between 2015 and 2022 can be found in Appendix 4.

### OPSS vs SHS chlamydia testing

SHS testing decreased as a proportion of all chlamydia testing from 43.8% in 2015 to 29.9% in 2022. Focusing on the comparison between OPSS and SHS testing services only (excluding all other in-person testing) in 2022, multivariable logistic regression indicated women were more likely than men to use OPSS (aOR 1.41 [1.40–1.42]) but the same associations as for OPSS vs all in-person testing were found for age group and IMD (Appendix 5). Compared to people in rural areas, Londoners were more likely to test via OPSS than SHS (OR 1.34 [1.32–1.35]) and people in other urban areas were less likely to do so (OR 0.88 [0.88–0.89]). There were some differences in use of OPSS between different regions. OPSS was associated with a lower likelihood of a positive diagnosis than SHS testing both before and after adjustment (OR 0.70 [0.70–0.71], aOR 0.69 [0.68–0.70]).

## Discussion

Online sexual health services are part of the changing sexual health economy and not a ‘bolt on’ or standalone service^30^ and our analysis situates OPSS within the overall trends for chlamydia testing and diagnosis across all testing services. In this retrospective cohort study, we found that although the overall number of chlamydia tests among men and women decreased during the COVID pandemic, and numbers in 2022 had not reached pre-COVID levels, there has been a shift towards testing via OPSS with the number and proportion of OPSS tests continuing to increase between 2015 and 2022. The reduction in testing and diagnoses at the start of the COVID-19 pandemic,^31^^,^^32^ with overall testing activity not returning to pre-COVID levels by 2022,^33^ and increasing proportion of home-based self-sampling compared to clinic-based testing over time^11^ is consistent with findings from other countries.

Globally, there is limited evidence of the impact of OPSS on equity of access to STI testing, particularly around literature comparing OPSS and in person testing.^34^ Groups including gay, bisexual and other men who have sex with men (GBMSM), black ethnic minorities, and young people aged 15–24 have disproportionately higher rates of STIs^2^ We were unable to examine testing behaviour for GBMSM compared to other men, but found that men overall were less likely to access chlamydia testing than women, and were proportionately more likely to test in SHSs and via OPSS compared to women. A national evaluation in Sweden similarly found that there was a higher uptake of online testing compared to clinic-based testing in men.^11^

Much of the testing by women occurred in general practice and other non-specialist in-person settings, which is related to the higher uptake of opportunistic chlamydia screening by women through the NCSP, leading to more testing in non-specialist settings, albeit with lower test-positivity. However, when focusing on SHSs only compared to OPSS, we found women were more likely than men to use OPSS. This distinction has not been previously highlighted in studies comparing SHSs only with OPSS, although further research into the effects of sexual orientation and sexual behaviour is required. This finding could be due to heightened awareness in women of the need to screen for chlamydia due to the NCSP programme,^35^ and because of awareness of the reproductive complications of chlamydia infection.

Other studies have found that those aged under 24 are less likely to access OPSS for chlamydia testing compared to older age groups,^18^ with evidence suggesting young people living at home prefer not to receive an STI testing kit in the post, due to concerns about privacy.^36^^,^^37^ It may also be related to their motivation for testing and understanding of asymptomatic infection. Young people are not always fully aware of the benefits of screening without symptoms^38^ and those reporting symptoms during an online triage are advised to test in-person at a SHS and, depending on the OPSS provider and nature of their symptoms, may not be eligible to receive a kit. There is no evidence that changes to NCSP in 2021 from testing all sexually active young people to focusing on women and other people with a womb and ovaries (those who experience the harms associated with chlamydia) had a notable impact on OPSS testing for chlamydia overall.^35^

A recent international review concluded that the beneficial impact on testing uptake with online STI interventions was less in historically marginalised populations.^34^ Our findings that those living in the most deprived areas were the least likely to test for chlamydia using OPSS is consistent with previous research.^14^^,^^15^^,^^18^^,^^20^ It is important to understand whether this indicates unmet need or whether other testing pathways are meeting the needs of this population. For example, chlamydia diagnoses were higher among young women living in the most deprived areas although testing activity was not.^39^

London had the largest proportion of OPSS use overall compared to other areas, and half of all London's chlamydia testing was via OPSS in 2022. These data show the dramatic impact of London's comprehensive OPSS service with near complete coverage.^40^^,^^41^ We found differences between the regions on use of OPSS which are complicated due to geographical differences in provision, which are not currently captured. A central repository which records details of OPSS services including region covered, and date of implementation would aid interpretation of these data.

Our data showed a drop in OPSS in the last quarter of 2022. The most recently published national statistics for chlamydia testing among young women show OPSS testing continued to decrease in 2023.^2^ This may be related to factors such as return to in-person testing and capping of online service provision and requires further investigation.

During this eight-year period, OPSS test positivity tended to decrease among men and women while SHS test positivity tended to increase. The highest SHS test positivity was in 2020, the year when restrictions in access to face-to-face services in SHSs were greatest. There have been limited evaluations comparing test positivity in clinic-based and online settings, but other studies have also found that test positivity is higher in clinic-based settings.^11^^,^^15^^,^^16^ With the overall reduction in testing between 2020 and 2022 compared to pre-pandemic, it is unclear if the fall in diagnoses is due to a reduction in prevalence or an increase in unmet need.

The gender differences in test positivity may be explained by the fact that chlamydia is more likely to be symptomatic in men who would therefore be channelled to in-person services while SHSs have increasingly restricted their offer of testing to symptomatic people and those with more complex needs. In addition, there is a higher uptake of chlamydia screening among women via the NCSP.

The rationale for screening for STIs, particularly frequency of screening for chlamydia and gonorrhoea in GBMSM, has been questioned in recent years. A recent review article highlights the lack of evidence that screening for chlamydia and gonorrhoea reduces their prevalence and associated complications, whilst highlighting the potential for increased antimicrobial resistance (AMR) due to increased consumption of antibacterials in certain key populations.^42^ Although we were unable to evaluate testing behaviour for GBMSM, the inequity found within this analysis could also be the case for this population. However, the health impact of this would be less clear given the lack of significant health outcomes of chlamydia in people assigned male at birth and concerns about the impact of screening on AMR.^42^

A key strength of our study is the use of data collected for two mandatory STI surveillance systems which undergo rigorous cleaning to ensure the best possible quality.^43^ Previous studies of OPSS are often based on single SHSs,^18^ while the combined GUMCAD and CTAD dataset provides data on all publicly funded chlamydia testing in England. We provide a comprehensive analysis of changes in chlamydia testing across the entire population in all settings over time.

CTAD and GUMCAD data are depersonalised datasets which cannot be used to reveal anyone's identity—it is not therefore possible to link individuals between data sets or, to link people between different services within each data set. The unit of analysis was chlamydia testing within a 6-week episode and individuals may therefore be included multiple times. The finding that certain groups are more likely to use OPSS may be accentuated by repeated use among these users. At the same time, sexual health services with integrated OPSS services could not distinguish between SHS and OPSS testing when they submitted their returns to GUMCAD over the time period used in this analysis. Although this may result in a slight underestimation of OPSS use and mask differences in the uptake of OPSS by different groups, the proportion of this underestimation has been estimated and only affects data from 2020 (0.2%), 2021 (1.4%) and 2022 (2.4%).

The focus here is on chlamydia testing only, as CTAD provides the most complete data on use of OPSS for testing for any STI. Our analysis may function as a proxy for overall STI testing, assuming that chlamydia testing is always offered as part of routine STI testing. Incorporation of CTAD, however, limits the analysis to variables included within this dataset which are more limited than GUMCAD. We were unable, for example, to analyse by sexual orientation which is included in GUMCAD but not in CTAD. While testing guidelines differ according to sexual and lifestyle behaviours, and for those using HIV Pre-Exposure Prophylaxis (PrEP), which is measured in GUMCAD but not in CTAD, we were unable to adjust for this in our analysis to examine whether differences between men and women were driven primarily by these factors. There is a high degree of item non-response for ethnic group in CTAD (56.7% vs 6.4% in GUMCAD) and we were unable to analyse use of OPSS according to ethnicity, but we have used IMD data to provide a measure of equity of uptake given its strong correlation with ethnicity (ethnic minorities are over-represented in more deprived areas).

This analysis focuses on the uptake of chlamydia testing and test positivity in different settings, and we were unable to provide insights on the steps further along the care cascade including treatment outcomes due to the limitations of these datasets. This is addressed by analysing service-level data as part of the wider ASSIST project.^23^

Although 15-year-olds are not eligible for OPSS, we have included them in the youngest age group (15–19 years) to reduce the risk of deductive disclosure when cross-referring to other publications.^2^^,^^3^ This is likely to have little impact on the analysis, given that 15-year-olds represent only 0.6% of all tests during our study period.

We found variation in use of OPSS by sociodemographic characteristics (age group, gender and area level deprivation) and differences in positivity by testing mode and gender. Our findings demonstrate that different modes of testing need to be available in order to maintain access across population groups and to avoid inequity in access to care. These findings need to be taken into consideration when considering the implementation of OPSS within Europe. Further research is needed to ensure available testing pathways meet the needs of all populations.

## Contributors

The study was conceived by FB, CE, JG and JS, and designed by FB, CE, JG, AH, HM and JS. Data management was conducted by SB, AKH, AH, TS and AT. AH and AKH have directly accessed and verified the underlying data. Data were analysed by AH, with input from SB, FB, AC, JG, HM, JS, OS and AT. The manuscript was drafted by AH. All authors contributed to the interpretation of findings and critically reviewed the manuscript. All authors have read and approved the final version, and take responsibility for the decision to submit the manuscript.

## Data sharing statement

Applications for requests to access relevant anonymised data included in this study should be submitted to the UK Health Security Agency Office for data release at: https://www.gov.uk/government/publications/accessing-ukhsa-protected-data/accessing-ukhsa-protected-data.

## Declaration of interests

The following authors declare competing interests:

FB has received speakers' fees and an institutional grant from Gilead Sciences Ltd., as well as other institutional grants from the NIHR. JG has received institutional grants from the NIHR.; JS is co-chair of the BASHH bacterial STI special interest group and has received travel support from BHIVA & BASHH for meetings associated with this work.

The other authors declare no potential conflicts of interest with respect to the research, authorship, and/or publication of this article.

## Appendix A Supplementary data

Appendix

Gibbs_ASSIST protocol
