# Supplementary material for: Uptake, positivity, and equity of online postal self-sampling for chlamydia testing in England: a retrospective cohort study
Source: Lancet Reg Health Eur. 2025 Aug 15;56:101412. doi: 10.1016/j.lanepe.2025.101412 (PMC12392761; doi:10.1016/j.lanepe.2025.101412)
Supplement: Appendix [file mmc1.docx]

**Appendix 1. Number of chlamydia tests and positivity by mode of testing and gender, 2015 – 2022**

|  | Year | Sexual Health Services ^ | Community ^ | GP ^ | Pharmacy ^ | TOP^±^ services ^ | OPSS ^ | Other ^ | Unknown ^ | Total ^ |
| --- | --- | --- | --- | --- | --- | --- | --- | --- | --- | --- |
| **Men** | 2015 | 692,873 (8∙2) | 107,723 (9∙6) | 99,515 (6∙2) | 4,639 (11∙7) | 108 (8∙3) | 27,557 (9∙3) | 119,686 (6∙0) | 5,996 (6∙8) | 1,058,097 (8∙0) |
|  | 2016 | 688,782 (8∙5) | 80,938 (10∙3) | 95,744 (6∙4) | 4,353 (10∙4) | 53 (3∙8) | 49,312 (8∙3) | 99,927 (6∙5) | 8,301 (8∙3) | 1,027,410 (8∙3) |
|  | 2017 | 691,355 (9∙0) | 41,254 (11∙7) | 95,850 (6∙8) | 4,172 (10∙5) | 64 (7∙8) | 68,302 (7∙8) | 83,158 (6∙9) | 7,983 (8∙1) | 992,138 (8∙7) |
|  | 2018 | 706,377 (9∙5) | 36,255 (11∙6) | 92,603 (6∙5) | 4,182 (12∙5) | 25 (8∙0) | 137,428 (7∙1) | 88,452 (6∙6) | 5,242 (6∙1) | 1,070,564 (8∙8) |
|  | 2019 | 712,195 (10∙0) | 31,447 (11∙9) | 88,803 (6∙0) | 4,052 (11∙9) | 92 (6∙5) | 194,234 (6∙6) | 93,683 (6∙6) | 7,274 (6∙2) | 1,131,780 (8∙9) |
|  | 2020 | 341,215 (11∙0) | 10,980 (12∙3) | 55,061 (5∙4) | 2,351 (13∙0) | 31 (6∙5) | 309,614 (7∙3) | 62,835 (7∙0) | 5,473 (7∙3) | 787,560 (8∙9) |
|  | 2021 | 345,731 (10∙2) | 11,007 (10∙6) | 53,491 (4∙3) | 1,828 (12∙6) | 62 (6∙5) | 390,763 (6∙7) | 80,172 (6∙5) | 3,930 (5∙8) | 886,984 (7∙9) |
|  | 2022 | 406,161 (10∙6) | 11,353 (10∙7) | 60,859 (5∙3) | 1,467 (13∙4) | 175 (10∙3) | 454,596 (7∙5) | 81,270 (7∙2) | 3,737 (7∙6) | 1,019,618 (8∙6) |
|  |  |  |  |  |  |  |  |  |  |  |
|  | Total men | 4,584,689 (9∙4) | 330,957 (10∙7) | 641,926 (6) | 27,044 (11∙7) | 610 (7∙9) | 1,631,806 (7∙2) | 709,183 (6∙6) | 47,936 (7∙1) | 7,974,151 (8∙5) |
|  |  |  |  |  |  |  |  |  |  |  |
| **Women** | 2015 | 797,143 (6∙4) | 271,848 (6∙9) | 807,585 (2∙7) | 20,327 (5∙5) | 47,883 (3∙9) | 60,367 (7∙4) | 333,375 (4∙3) | 37,362 (3∙1) | 2,375,890 (4∙8) |
|  | 2016 | 807,986 (6∙8) | 191,326 (7∙1) | 756,565 (2∙8) | 12,086 (8∙1) | 53,968 (3∙8) | 107,904 (7∙1) | 284,541 (4∙5) | 48,578 (3∙5) | 2,262,954 (5∙1) |
|  | 2017 | 815,848 (7∙2) | 106,130 (8∙0) | 745,089 (3∙0) | 11,127 (8∙8) | 53,196 (4∙4) | 132,492 (7∙4) | 252,233 (4∙6) | 43,645 (3∙8) | 2,159,760 (5∙4) |
|  | 2018 | 818,265 (7∙6) | 83,182 (8∙5) | 713,250 (2∙9) | 10,569 (9∙2) | 46,114 (3∙8) | 246,889 (6∙6) | 259,687 (4∙3) | 30,712 (3∙2) | 2,208,668 (5∙5) |
|  | 2019 | 818,596 (7∙7) | 78,552 (8∙2) | 683,256 (2∙7) | 10,134 (9∙6) | 54,933 (4∙3) | 338,594 (6∙1) | 268,470 (4∙3) | 40,032 (3∙2) | 2,292,567 (5∙4) |
|  | 2020 | 407,903 (8∙0) | 28,946 (7∙8) | 481,680 (2∙3) | 6,804 (9∙7) | 22,596 (4∙9) | 517,825 (5∙9) | 193,890 (4∙2) | 33,141 (3∙2) | 1,692,785 (5∙2) |
|  | 2021 | 401,579 (7∙2) | 27,102 (6∙0) | 523,578 (1∙9) | 5,679 (8∙4) | 12,418 (4∙0) | 642,506 (5∙2) | 246,938 (3∙6) | 34,002 (2∙4) | 1,893,802 (4∙5) |
|  | 2022 | 420,760 (7∙9) | 24,601 (7∙2) | 535,858 (2∙2) | 4,315 (10∙6) | 15,018 (5∙2) | 702,867 (6∙1) | 225,869 (4∙2) | 23,320 (3∙2) | 1,952,608 (5∙2) |
|  |  |  |  |  |  |  |  |  |  |  |
|  | Total women | 5,288,080 (7∙3) | 811,687 (7∙4) | 5,246,861 (2∙6) | 81,041 (8∙1) | 306,126 (4∙2) | 2,749,444 (6) | 2,065,003 (4∙3) | 290,792 (3∙2) | 16,839,034 (5∙1) |

^ Number of chlamydia tests (Positivity) ±TOP Termination of Pregnancy

**Appendix 2– GEE sensitivity analysis of mode of testing (OPSS vs in-person) by sociodemographic factors and testing behaviour (2015-22)**

|  | **N of tests** | **In-person %** | **OPSS %** ^∞^ | **OR** ^∞^ | **[CI]** | **aOR** ^∞^ | **[CI]** |
| --- | --- | --- | --- | --- | --- | --- | --- |
| **Gender ^§^** |  |  |  |  |  |  |  |
| Men | 7,926,215 | 6,294,409 (79·4) | 1,631,806 (20·6) | Ref |  | Ref |  |
| Women | 16,548,242 | 13,798,798 (83·4) | 2,749,444 (16·6) | 0.68 | [0.68-0.68] | 0.68 | [0.67-0.68] |
| Other / Unknown | 350,092 | 322,473 (92·1) | 27,619 (7·9) | - | - | - | - |
| **Age group** |  |  |  |  |  |  |  |
| 15 – 19 years | 3,090,817 | 2,630,050 (85·1) | 460,767 (14·9) | Ref |  | Ref |  |
| 20 – 24 years | 6,655,847 | 5,038,471 (75·7) | 1,617,376 (24·3) | 1.84 | [1.83-1.84] | 1.53 | [1.52-1.53] |
| 25 – 34 years | 8,727,830 | 7,101,988 (81·4) | 1,625,842 (18·6) | 1.34 | [1.33-1.34] | 0.82 | [0.82-0.82] |
| 35 – 44 years | 3,954,544 | 3,465,305 (87·6) | 489,239 (12·4) | 0.82 | [0.81-0.82] | 0.46 | [0.46-0.46] |
| 45 – 64 years | 2,238,058 | 2,032,608 (90·8) | 205,450 (9·2) | 0.59 | [0.59-0.59] | 0.33 | [0.32-0.33] |
| 65 years & over | 157,453 | 147,258 (93·5) | 10,195 (6·5) | 0.41 | [0.40-0.41] | 0.20 | [0.20-0.21] |
| **IMD ^#^** |  |  |  |  |  |  |  |
| 1 – most deprived | 5,382,701 | 4,577,238 (85·0) | 805,463 (15·0) | Ref |  | Ref |  |
| 2 | 5,948,222 | 4,811,457 (80·9) | 1,136,765 (19·1) | 1.35 | [1.35-1.36] | 1.17 | [1.17-1.17] |
| 3 | 5,010,645 | 4,068,197 (81·2) | 942,448 (18·8) | 1.30 | [1.30-1.31] | 1.17 | [1.17-1.18] |
| 4 | 4,052,141 | 3,309,907 (81·7) | 742,234 (18·3) | 1.25 | [1.24-1.25] | 1.24 | [1.24-1.25] |
| 5 – least deprived | 3,602,215 | 2,991,445 (83) | 610,770 (17) | 1.13 | [1.12=1.13] | 1.16 | [1.16-1.17] |
| Unknown | 828,625 | 657,436 (79·3) | 171,189 (20·7) | - | - | - | - |
| **Region of residency** |  |  |  |  |  |  |  |
| London | 6,820,758 | 5,178,384 (75·9) | 1,642,374 (24·1) | Ref |  | Ref |  |
| North East | 988,416 | 886,441 (89·7) | 101,975 (10·3) | 0.34 | [0.33-0.34] | 0.32 | [0.31-0.32] |
| North West | 2,865,396 | 2,582,481 (90·1) | 282,915 (9·9) | 0.32 | [0.31-0.32] | 0.32 | [0.32-0.32] |
| Yorkshire & Humber | 2,361,443 | 2,002,946 (84·8) | 358,497 (15·2) | 0.51 | [0.51-0.52] | 0.51 | [0.51-0.52] |
| East Midlands | 1,888,801 | 1,551,567 (82·1) | 337,234 (17·9) | 0.61 | [0.61-0.61] | 0.62 | [0.62-0.63] |
| East of England | 2,333,138 | 1,884,355 (80·8) | 448,783 (19·2) | 0.68 | [0.68-0.68] | 0.69 | [0.69-0.70] |
| West Midlands | 1,985,554 | 1,682,149 (84·7) | 303,405 (15·3) | 0.54 | [0.54-0.54] | 0.56 | [0.55-0.56] |
| South East | 3,100,684 | 2,631,448 (84·9) | 469,236 (15·1) | 0.53 | [0.53-0.54] | 0.53 | [0.52-0.53] |
| South West | 2,157,507 | 1,774,966 (82·3) | 382,541 (17·7) | 0.62 | [0.62-0.62] | 0.65 | [0.65-0.65] |
| Unknown | 322,852 | 240,943 (74·6) | 81,909 (25·4) | - | - | - | - |
| **London-rural-urban*** |  |  |  |  |  |  |  |
| Rural | 2,487,943 | 2,104,217 (84·6) | 383,726 (15·4) | Ref |  | Ref |  |
| London | 6,816,179 | 5,175,123 (75·9) | 1,641,056 (24·1) | 1.93 | [1.92-1.93] | 1.14 | [1.14-1.15] |
| Other urban | 14,818,621 | 12,563,027 (84·8) | 2,255,594 (15·2) | 1.01 | [1.00-1.01] |  |  |
| Unknown | 701,806 | 573,313 (81·7) | 128,493 (18·3) | - | - | - | - |
| **Year of testing** |  |  |  |  |  |  |  |
| 2015 | 3,416,842 | 3,328,698 (97·4) | 88,144 (2·6) | Ref |  | Ref |  |
| 2016 | 3,268,821 | 3,110,384 (95·2) | 158,437 (4·8) | 1.92 | [1.90-1.93] | 1.91 | [1.90-1.92] |
| 2017 | 3,128,445 | 2,925,538 (93·5) | 202,907 (6·5) | 2.66 | [2.64-2.67] | 2.69 | [2.67-2.71] |
| 2018 | 3,273,977 | 2,887,124 (88·2) | 386,853 (11·8) | 4.98 | [4.95-5.02] | 5.02 | [4.99-5.05] |
| 2019 | 3,414,926 | 2,879,451 (84·3) | 535,475 (15·7) | 6.86 | [6.82-6.91] | 6.91 | [6.87-6.96] |
| 2020 | 2,469,708 | 1,639,427 (66·4) | 830,281 (33·6) | 17.60 | [17.49-17.71] | 18.31 | [18.19-18.42] |
| 2021 | 2,804,913 | 1,766,969 (63) | 1,037,944 (37) | 20.43 | [20.30-20.56] | 22.40 | [22.27-22.54] |
| 2022 | 3,046,917 | 1,878,089 (61·6) | 1,168,828 (38·4) | 21.15 | [21.02-21.28] | 23.77 | [23.62-23.92] |
| **Positive diagnosis** |  |  |  |  |  |  |  |
| No | 23,267,663 | 19,143,739 (82·3) | 4,123,924 (17·7) | Ref |  | Ref |  |
| Yes | 1,556,886 | 1,271,941 (81·7) | 284,945 (18·3) | 1.09 | [1.09-1.10] | 0.93 | [0.93-0.94] |

**Appendix 3 – Mode of testing (OPSS vs in-person) by sociodemographic factors and testing behaviour (2022)**

|  | **N** | **In-person %** | **OPSS %** ^∞^ | **OR** ^∞^ | **[CI]** | **aOR** ^∞^ | **[CI]** |
| --- | --- | --- | --- | --- | --- | --- | --- |
| **Gender ^§^** |  |  |  |  |  |  |  |
| Men | 1,015,881 | 561,285 (55.3) | 454,596 (44.7) | Ref |  | Ref |  |
| Women | 1,929,288 | 1,226,421 (63.6) | 702,867 (36.4) | 0·71 | [0·70-0·71] | 0·71 | [0·71-0·71] |
| Other / Unknown | 101,748 | 90,383 (88.8) | 11,365 (11.2) | - | - | - | - |
| **Age group** |  |  |  |  |  |  |  |
| 15 – 19 years | 264,988 | 172,948 (65.3) | 92,040 (34.7) | Ref |  | Ref |  |
| 20 – 24 years | 730,534 | 379,696 (52) | 350,838 (48) | 1·74 | [1·72-1·75] | 1·58 | [1·57-1·60] |
| 25 – 34 years | 1,161,649 | 673,729 (58) | 487,920 (42) | 1·36 | [1·35-1·37] | 1·08 | [1·07-1·09] |
| 35 – 44 years | 556,003 | 391,297 (70.4) | 164,706 (29.6) | 0·79 | [0·78-0·80] | 0·62 | [0·61-0·62] |
| 45 – 64 years | 309,060 | 239,524 (77.5) | 69,536 (22.5) | 0·55 | [0·54-0·55] | 0·42 | [0·41-0·42] |
| 65 years & over | 24,683 | 20,895 (84.7) | 3,788 (15.3) | 0·34 | [0·33-0·35] | 0·25 | [0·25-0·26] |
| **IMD ^#^** |  |  |  |  |  |  |  |
| 1 – most deprived | 641,768 | 421,272 (65.6) | 220,496 (34.4) | Ref |  | Ref |  |
| 2 | 751,767 | 449,158 (59.7) | 302,609 (40.3) | 1·29 | [1·28-1·30] | 1·12 | [1·11-1·12] |
| 3 | 612,973 | 372,946 (60.8) | 240,027 (39.2) | 1·23 | [1·22-1·24] | 1·12 | [1·11-1·13] |
| 4 | 476,664 | 287,729 (60.4) | 188,935 (39.6) | 1·25 | [1·24-1·26] | 1·25 | [1·24-1·26] |
| 5 – least deprived | 423,836 | 273,308 (64.5) | 150,528 (35.5) | 1·05 | [1·04-1·06] | 1·11 | [1·10-1·12] |
| Unknown | 139,909 | 73,676 (52.7) | 66,233 (47.3) | - |  | - | - |
| **Region of residency** |  |  |  |  |  |  |  |
| London | 901,830 | 456,751 (50.6) | 445,079 (49.4) | Ref |  | Ref |  |
| North East | 117,057 | 90,150 (77) | 26,907 (23) | 0·31 | [0·30-0·31] | 0·32 | [0·31-0·32] |
| North West | 335,554 | 242,369 (72.2) | 93,185 (27.8) | 0·39 | [0·39-0·40] | 0·44 | [0·44-0·45] |
| Yorkshire & Humber | 286,404 | 206,888 (72.2) | 79,516 (27.8) | 0·39 | [0·39-0·40] | 0·41 | [0·40-0·41] |
| East Midlands | 249,950 | 167,903 (67.2) | 82,047 (32.8) | 0·50 | [0·50-0·51] | 0·54 | [0·54-0·55] |
| East of England | 269,736 | 132,496 (49.1) | 137,240 (50.9) | 1·06 | [1·05-1·07] | 1·21 | [1·20-1·22] |
| West Midlands | 232,174 | 145,176 (62.5) | 86,998 (37.5) | 0·61 | [0·61-0·62] | 0·65 | [0·64-0·66] |
| South East | 356,672 | 253,894 (71.2) | 102,778 (28.8) | 0·42 | [0·41-0·42] | 0·42 | [0·42-0·43] |
| South West | 235,121 | 151,408 (64.4) | 83,713 (35.6) | 0·57 | [0·56-0·57] | 0·63 | [0·63-0·64] |
| Unknown | 62,419 | 31,054 (49.8) | 31,365 (50.2) | - | - | - | - |
| **London-rural-urban** |  |  |  |  |  |  |  |
| Rural | 287,944 | 192,944 (67.0) | 95,000 (33.0) | Ref |  | Ref |  |
| London | 901,313 | 456,540 (50.7) | 444,773 (49.3) | 1·98 | [1·96-2·00] | 1·12 | [1·12-1·14] |
| Other urban | 1,738,753 | 1,159,707 (66.7) | 579,046 (33.3) | 1·01 | [1·01-1·02] |  |  |
| Unknown | 118,907 | 68,898 (57.9) | 50,009 (42.1) | - | - | - | - |
| **Positive diagnosis** |  |  |  |  |  |  |  |
| No | 2,850,275 | 1,759,231 (61.7) | 1,091,044 (38.3) | Ref |  | Ref |  |
| Yes | 196,642 | 118,858 (60.4) | 77,784 (39.6) | 1·06 | [1·05-1·07] | 0·93 | [0·92-0·93] |

**Appendix 4 – Diagnosis by sociodemographic factors and testing behaviour (2015-22)**

|  | N | Negative (%) | Positive (%) ∞ | **OR** ^∞^ | **[CI]** | **aOR** ^∞^ | **[CI]** |
| --- | --- | --- | --- | --- | --- | --- | --- |
| **Gender ^§^** |  |  |  |  |  |  |  |
| Men | 7,974,151 | 7,296,490 (91·5) | 677,661 (8·5) | Ref |  |  |  |
| Women | 16,839,034 | 15,975,468 (94·9) | 863,566 (5·1) | 0·58 | [0·58-0·58] | 0·52 | [0·52-0·53] |
| Other / Unknown | 358,734 | 329,878 (92·0) | 28,856 (8·0) | - | - | - | - |
| **Age group** |  |  |  |  |  |  |  |
| 15 – 19 years | 3,138,192 | 2,781,203 (88·6) | 356,989 (11·4) | Ref |  |  |  |
| 20 – 24 years | 6,730,227 | 6,138,187 (91·2) | 592,040 (8·8) | 0·75 | [0·75-0·75] | 0·72 | [0·71-0·72] |
| 25 – 34 years | 8,846,591 | 8,407,358 (95·0) | 439,233 (5·0) | 0·41 | [0·41-0·41] | 0·37 | [0·37-0·37] |
| 35 – 44 years | 4,022,392 | 3,902,312 (97·0) | 120,080 (3·0) | 0·24 | [0·24-0·24] | 0·22 | [0·22-0·22] |
| 45 – 64 years | 2,274,291 | 2,216,125 (97·4) | 58,166 (2·6) | 0·20 | [0·20-0·21] | 0·18 | [0·18-0·18] |
| 65 years & over | 160,226 | 156,651 (97·8) | 3,575 (2·2) | 0·18 | [0·17-0·18] | 0·14 | [0·13-0·14] |
| **IMD ^#^** |  |  |  |  |  |  |  |
| 1 – most deprived | 5,470,769 | 5,074,428 (92·8) | 396,341 (7·2) | Ref |  |  |  |
| 2 | 6,032,494 | 5,639,059 (93·5) | 393,435 (6·5) | 0·89 | [0·89-0·90] | 0·91 | [0·91-0·92] |
| 3 | 5,067,826 | 4,763,672 (94·0) | 304,154 (6·0) | 0·82 | [0·81-0·82] | 0·83 | [0·83-0·84] |
| 4 | 4,106,988 | 3,879,934 (94·5) | 227,054 (5·5) | 0·75 | [0·75-0·75] | 0·76 | [0·76-0·77] |
| 5 – least deprived | 3,642,786 | 3,452,719 (94·8) | 190,067 (5·2) | 0·70 | [0·70-0·71] | 0·70 | [0·70-0·71] |
| Unknown | 851,056 | 792,024 (93·1) | 59,032 (6·9) | - | - | - | - |
| **Region of residency** |  |  |  |  |  |  |  |
| London | 6,858,584 | 6,452,132 (94·1) | 406,452 (5·9) | 0·89 | [0·89-0·90] | 1·00 | [0·99-1·01] ^++^ |
| North East | 1,028,762 | 961,005 (93·4) | 67,757 (6·6) | Ref |  |  |  |
| North West | 2,972,657 | 2,764,628 (93·0) | 208,029 (7·0) | 1·07 | [1·06-1·08] | 1·07 | [1·07-1·09] |
| Yorkshire & Humber | 2,368,262 | 2,214,379 (93·5) | 153,883 (6·5) | 0·99 | [0·98-0·99] ^+^ | 1·05 | [1·04-1·06] |
| East Midlands | 1,916,118 | 1,793,327 (93·6) | 122,791 (6·4) | 0·97 | [0·96-0·98] | 1·07 | [1·06-1·08] |
| East of England | 2,339,158 | 2,207,417 (94·4) | 131,741 (5·6) | 0·85 | [0·84-0·85] | 0·95 | [0·95-0·96] |
| West Midlands | 2,001,552 | 1,863,889 (93·1) | 137,663 (6·9) | 1·05 | [1·04-1·06] | 1·09 | [1·08-1·10] |
| South East | 3,195,993 | 3,006,004 (94·1) | 189,989 (5·9) | 0·90 | [0·89-0·90] | 1·03 | [1·02-1·04] |
| South West | 2,162,879 | 2,037,361 (94·2) | 125,518 (5·8) | 0·87 | [0·87-0·88] | 0·95 | [0·94-0·96] |
| Unknown | 327,954 | 301,694 (92·0) | 26,260 (8·0) | - | - | - | - |
| **London-rural-urban** |  |  |  |  |  |  |  |
| Rural | 2,529,140 | 2,392,465 (94·6) | 136,675 (5·4) | Ref |  |  |  |
| London | 6,853,972 | 6,447,768 (94·1) | 406,204 (5·9) | 1·18 | [1·17-1·19] | 1·08 | [1·08-1·09] |
| Other urban | 15,065,474 | 14,089,842 (93·5) | 975,632 (6·5) |  |  |  |  |
| Unknown | 723,333 | 671,761 (92·9) | 51,572 (7·1) | - | - | - | - |
| **Year of testing** |  |  |  |  |  |  |  |
| 2015 | 3,460,881 | 3,260,147 (94·2) | 200,734 (5·8) | Ref |  |  |  |
| 2016 | 3,329,283 | 3,126,530 (93·9) | 202,753 (6·1) | 1·05 | [1·05-1·06] | 1·09 | [1·08-1·09] |
| 2017 | 3,182,064 | 2,978,145 (93·6) | 203,919 (6·4) | 1·11 | [1·11-1·12] | 1·18 | [1·17-1·19] |
| 2018 | 3,310,084 | 3,092,606 (93·4) | 217,478 (6·6) | 1·14 | [1·13-1·15] | 1·24 | [1·23-1·25] |
| 2019 | 3,463,456 | 3,235,490 (93·4) | 227,966 (6·6) | 1·14 | [1·14-1·15] | 1·27 | [1·26-1·28] |
| 2020 | 2,508,722 | 2,348,422 (93·6) | 160,300 (6·4) | 1·11 | [1·10-1·12] | 1·28 | [1·27-1·29] |
| 2021 | 2,843,026 | 2,683,803 (94·4) | 159,223 (5·6) | 0·96 | [0·96-0·97] | 1·15 | [1·15-1·16] |
| 2022 | 3,074,403 | 2,876,693 (93·6) | 197,710 (6·4) | 1·12 | [1·11-1·12] | 1·35 | [1·34-1·36] |
| **Testing mode** |  |  |  |  |  |  |  |
| In-person | 20,415,680 | 19,143,739 (93·8) | 1,271,941 (6·2) | Ref |  |  |  |
| OPSS | 4,408,869 | 4,123,924 (93·5) | 284,945 (6·5) | 1·04 | [1·04-1·04] | 0·88 | [0·87-0·88] |
| Unknown | 347,370 | 334,173 (96·2) | 13,197 (3·8) | - | - | - | - |
| **Testing mode (complete)** |  |  |  |  |  |  |  |
| Sexual health services ^±^ | 10,108,131 | 9,270,871 (91·7) | 837,260 (8·3) | - | - | - | - |
| OPSS | 4,408,869 | 1,068,993 (91·6) | 97,592 (8·4) | - | - | - | - |
| Community | 1,166,585 | 5,732,049 (97·0) | 176,443 (3·0) | - | - | - | - |
| GP | 5,908,492 | 99,108 (91·0) | 9,849 (9·0) | - | - | - | - |
| Pharmacy | 108,957 | 300,667 (95·8) | 13,099 (4·2) | - | - | - | - |
| TOP services ^±^ | 313,766 | 4,123,924 (93·5) | 284,945 (6·5) | - | - | - | - |
| Other | 2,809,749 | 2,672,051 (95·1) | 137,698 (4·9) | - | - | - | - |
| Unknown | 347,370 | 334,173 (96·2) | 13,197 (3·8) | - | - | - | - |

**Appendix 5 – Mode of testing (OPSS vs SHS) by sociodemographic factors and testing behaviour (2022)**

|  | **N** | **Sexual health services %** | **OPSS %** ^∞^ | **OR** ^∞^ | **[CI]** | **aOR** ^∞^ | **[CI]** |
| --- | --- | --- | --- | --- | --- | --- | --- |
| **Gender ^§^** |  |  |  |  |  |  |  |
| Men | 860,757 | 406,191 (47·2) | 454,566 (52·8) | Ref |  | Ref |  |
| Women | 1,123,627 | 420,798 (37·5) | 702,829 (62·6) | 1·49 | [1·48-1·50] | 1·41 | [1·40-1·42] |
| Other / Unknown | 96,133 | 84,770 (88·2) | 11,363 (11·8) | - | - | - | - |
| **Age group** |  |  |  |  |  |  |  |
| 15 – 19 years | 179,732 | 87,691 (48·8) | 92,041 (51·2) | Ref |  | Ref |  |
| 20 – 24 years | 549,858 | 198,994 (36·2) | 350,864 (63·8) | 1·68 | [1·66-1·70] | 1·60 | [1·58-1·62] |
| 25 – 34 years | 823,966 | 336,013 (40·8) | 487,953 (59·2) | 1·38 | [1·37-1·40] | 1·23 | [1·22-1·24] |
| 35 – 44 years | 335,197 | 170,481 (50·9) | 164,716 (49·1) | 0·92 | [0·91-0·93] | 0·82 | [0·81-0·83] |
| 45 – 64 years | 178,301 | 108,764 (61·0) | 69,537 (39·0) | 0·61 | [0·60-0·62] | 0·57 | [0·56-0·57] |
| 65 years & over | 13,463 | 9,675 (71·9) | 3,788 (28·1) | 0·37 | [0·36-0·39] | 0·38 | [0·36-0·39] |
| **IMD ^#^** |  |  |  |  |  |  |  |
| 1 – most deprived | 432,225 | 211,747 (49·0) | 220,478 (51·0) | Ref |  | Ref |  |
| 2 | 534,688 | 232,055 (43.4) | 302,633 (56·6) | 1·25 | [1·24-1·26] | 1·14 | [1·13-1·15] |
| 3 | 420,497 | 180,477 (42.9) | 240,020 (57·1) | 1·28 | [1·27-1·29] | 1·18 | [1·17-1·19] |
| 4 | 327,587 | 138,668 (42.3) | 188,919 (57·7) | 1·31 | [1·30-1·32] | 1·27 | [1·26-1·29] |
| 5 – least deprived | 274,025 | 123,503 (45.1) | 150,522 (54·9) | 1·17 | [1·16-1·18] | 1·18 | [1·17-1·20] |
| Unknown | 91,495 | 25,262 (27.6) | 65,318 (71·4) | - | - | - | - |
| **Region of residency** |  |  |  |  |  |  |  |
| London | 717,599 | 272,544 (38·0) | 445,055 (62)·0 | Ref |  | Ref |  |
| North East | 69,870 | 42,963 (61·5) | 26,907 (38·5) | 0·38 | [0·38-0·39] | 0·39 | [0·38-0·40] |
| North West | 216,167 | 122,977 (56·9) | 93,190 (43·1) | 0·46 | [0·46-0·47] | 0·55 | [0·55-0·56] |
| Yorkshire & Humber | 153,211 | 73,694 (48·1) | 79,517 (51·9) | 0·66 | [0·65-0·67] | 0·62 | [0·61-0·63] |
| East Midlands | 140,282 | 58,231 (41·5) | 82,051 (58·5) | 0·86 | [0·85-0·87] | 0·92 | [0·90-0·93] |
| East of England | 189,590 | 52,346 (27.6) | 137,244 (72·4) | 1·61 | [1·59-1·62] | 1·84 | [1·81-1·86] |
| West Midlands | 164,867 | 77,867 (47·2) | 87,000 (52·8) | 0·68 | [0·68-0·69] | 0·68 | [0·68-0·69] |
| South East | 233,841 | 131,068 (56·1) | 102,773 (·0) | 0·48 | [0·48-0·48] | 0·46 | [0·45-0·46] |
| South West | 138,969 | 55,254 (39·8) | 83,715 (60·2) | 0·93 | [0·92-0·94] | 1·08 | [1·07-1·09] |
| Unknown | 56,121 | 24,755 (44·1) | 31,366 (55·9) | - | - | - | - |
| **London-rural-urban** |  |  |  |  |  |  |  |
| Rural | 172,838 | 77,846 (45·0) | 94,992 (55) | Ref |  | Ref |  |
| London | 717,173 | 272,382 (38·0) | 444,791 (62) | 1·34 | [1·32-1·35] | 1·04 | [1·03-1·05] |
| Other urban | 1,115,239 | 536,207 (48·1) | 579,032 (51.9) | 0·88 | [0·88-0·89] |  |  |
| Unknown | 75,267 | 25,260 (33·6) | 50,007 (66.4) | - | - | - | - |
| **Positive diagnosis** |  |  |  |  |  |  |  |
| No | 1,918,939 | 827,830 (43·1) | 1,091,109 (56.9) | Ref |  | Ref |  |
| Yes | 161,578 | 83,794 (51·9) | 77,784 (48.1) | 0·70 | [0·70-0·71] | 0·69 | [0·68-0·70] |
